# PET Study of Sphingosine-1-phosphate Receptor 1 Expression in Response to *S. aureus* Infection

**DOI:** 10.1155/2021/9982020

**Published:** 2021-10-04

**Authors:** Hao Jiang, Jiwei Gu, Haiyang Zhao, Sumit Joshi, Joel S. Perlmutter, Robert J. Gropler, Robyn S. Klein, Tammie L. S. Benzinger, Zhude Tu

**Affiliations:** ^1^Department of Radiology, Washington University School of Medicine, St Louis, MO 63110, USA; ^2^Department of Neuroscience, Neurology, Washington University School of Medicine, St Louis, MO 63110, USA; ^3^Department of Medicine, Washington University School of Medicine, St Louis, MO 63110, USA; ^4^Department of Pathology and Immunology, Washington University School of Medicine, St Louis, MO 63110, USA; ^5^Department of Neurological Surgery, Washington University School of Medicine, St Louis, MO 63110, USA

## Abstract

Sphingosine-1-phosphate receptor 1 (S1PR1) plays a crucial role in infectious diseases. Targeting S1PR1 provides protection against pathogens, such as influenza viruses. This study is aimed at investigating S1PR1 in response to bacterial infection by assessing S1PR1 expression in *S. aureus*-infected mice. A rodent local muscle bacterial infection model was developed by injecting *S. aureus* to the lower hind limb of Balb/c mice. The changes of S1PR1 expression in response to bacterial infection and blocking treatment were assessed using ex vivo biodistribution and *in vivo* positron emission tomography (PET) after intravenous injection of an S1PR1-specific radiotracer [^18^F]TZ4877. The specificity of [^18^F]TZ4877 was assessed using S1PR1-specific antagonist, NIBR-0213, and S1PR1-specific DsiRNA pretreated the animals. Immunohistochemical studies were performed to confirm the increase of S1PR1 expression in response to infection. *Ex vivo* biodistribution data showed that the uptake of [^18^F]TZ4877 was increased 30.6%, 54.3%, 74.3%, and 115.3% in the liver, kidney, pancreas, and thymus of the infected mice, respectively, compared to that in normal control mice, indicating that S1PR1 is involved in the early immune response to bacterial infection. NIBR-0213 or S1PR1-specific DsiRNA pretreatment reduced the tissue uptake of [^18^F]TZ4877, suggesting that uptake of [^18^F]TZ4877 is specific. Our PET/CT study data also confirmed that infected mice have increased [^18^F]TZ4877 uptake in several organs comparing to that in normal control mice. Particularly, compared to control mice, a 39% increase of [^18^F]TZ4877 uptake was observed in the infected muscle of *S. aureus* mice, indicating that S1PR1 expression was directly involved in the inflammatory response to infection. Overall, our study suggested that S1PR1 plays an important role in the early immune response to bacterial infection. The uptake of [^18^F]TZ4877 is tightly correlated with the S1R1 expression in response to *S. aureus* infection. PET with S1PR1-specific radiotracer [^18^F]TZ4877 could provide a noninvasive tool for detecting the early S1PR1 immune response to infectious diseases.

## 1. Introduction

*Staphylococcus aureus* (*S. aureus*) is a commensal human microbiota with approximately 30% of the human population which is colonized with *S. aureus* [1]. *S. aureus* often causes skin and soft tissue infections but can also cause pneumonia, bloodstream infections, endocarditis, and osteomyelitis [2–4]. To date, with the majority of studies focused on virus infection, particularly influenza, only little is known about the role of sphingosine-1-phosphate (S1P) and S1P receptors (S1PRs) in response to bacterial infection. Igawa and colleagues demonstrated that the expression of S1PR2 was elevated in *S. aureus*-treated human epidermal keratinocytes; they also showed that both S1PR1 and S1PR2 controlled the proinflammatory cytokine expression and secretion during the *S. aureus* invasion [5].

S1P binds to specific G protein-coupled S1P receptors 1-5 and triggers the S1P/S1PR pathway which plays a regulatory role in many pathophysiological processes, including angiogenesis [6], neurodegeneration [7], and immune response [8]. Intriguingly, it has been reported that S1P signaling via S1PRs is related to various aspects of inflammatory cell function. Accumulated evidence suggests that the S1P/S1PR pathway is impaired during infectious diseases. Furthermore, the S1P/S1PR pathway also plays an important role in infection-induced sepsis [9–11]. As a consequence, the S1P/S1PR pathway has been widely accepted as a therapeutic target in inflammatory and infection diseases [12]. Of note, several multiple sclerosis (MS) disease-modifying therapies targeting S1P/S1PRs are under investigation for treating coronavirus disease-2019 (COVID-19) [13, 14].

Out of the five S1PRs, S1PR1 is the most abundant S1P receptor and is widely studied in different diseases. It is ubiquitously expressed among tissues and is heavily involved in immune cell regulation. S1PR1 has been found to mediate functions in most immune cells and therefore plays a curial role in both innate and adaptive immune responses [15]. Previous studies suggest that S1PR1 is essential for lymphocyte recirculation by regulating lymphocyte egress from both the thymus and peripheral lymphoid organs [16, 17]; activation of S1PR1 inhibits the migration of T lymphocytes into different lymphatics and results in retention of T lymphocytes in nonlymphoid peripheral tissues [18]. S1PR1 also regulates the migration of B lymphocytes and osteoclasts [19, 20]. It was reported that S1PR1 plays an important role in the immune response to infectious diseases by regulating recruitment and trafficking of innate immune cells, macrophage polarization [21], and dendritic cell functions [22]. In addition, S1PR1 is considered to play a critical role in the development of sepsis [11]; for example, S1PR1-specific agonist SEW2871 can successfully protect against renal injury in a sepsis model [23]. Numerous studies show that S1PR1 expression or activation is tightly correlated with the inflammatory response to infectious diseases such as Newcastle disease virus infection [24], influenza A virus H1N1 [25], H9N2 infection [26], herpes simplex virus type 1 infection [27], *Pseudomonas aeruginosa* lung infection [28], and human immunodeficiency viruses HIV-1 infection [29]. It is also believed that host-directive therapy using modulators of S1PR1 and other S1PRs may be an effective treatment against severe infectious diseases, such as severe acute respiratory syndrome (SARS) and COVID-19 [30]. Despite the current understanding of S1PR1 in normal lymphocyte trafficking, the precise mechanisms of S1PR1 activation in response to pathogen-derived pathological infectious consequences remain not clear.

Our previous positron emission tomography (PET) studies with S1PR1-specific tracer demonstrated an increased expression of S1PR1 in rodent models of vascular inflammation and neuroinflammation [31, 32]. To evaluate the ability of S1PR1-specific tracer for the assessment of other inflammatory diseases, as well as to further understand the role of S1PR1 in response to pathogen infection, we performed ex vivo biodistribution, microPET, and immunohistochemistry staining studies to investigate S1PR1 expression on *S. aureus*-infected mice using our recently reported S1PR1-specific radiotracer [ [18]F]TZ4877 [33–35]. In our studies, we observed an increased uptake of [^18^F]TZ4877 in several organs in response to the infection; a 39% increase of [^18^F]TZ4877 uptake was also observed at the local hind limb muscle infected site. Our results indicated that the infection-induced increase of S1PR1 expression is tightly correlated with pathogen-derived inflammation. Our findings are consistent with other studies that S1PR1 plays a critical role in response to pathogen infection.

## 2. Materials and Methods

### 2.1. Animals

All animal experiments were conducted by following the Guidelines for the Care and Use of Research Animals under a research protocol approved by Washington University Institutional Animal Care and Use Committee (IACUC). Biodistribution and microPET/CT studies were conducted in Preclinical Imaging Facility at Washington University School of Medicine in St. Louis. For infection model, mice were injected with bacteria or PBS under 2–3% isoflurane in O_2_. For biodistribution study, mice were euthanized by cervical dislocation under anesthesia with 2–3% isoflurane in O_2_ and then, tissues of interest were collected. For microPET/CT studies, mice were euthanized by CO_2_ inhalation after use.

### 2.2. Radiosynthesis

**Figure 1:**
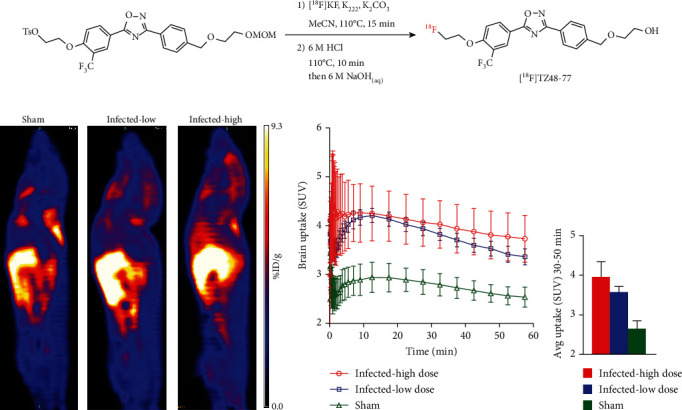
MicroPET imaging of S1PR1 activity in *S aureus*-infected mice. (a) Radiosynthesis of S1PR1-specific radiotracer, [^18^F]TZ4877; (b) representative sagittal microPET images of [^18^F]TZ4877 in mice. Comparing with sham mice, the tracer uptake was significantly higher in the infected mice, and the increased uptake of the tracer showed *S aureus* dose dependent; (c) the tracer uptake in the brain was quantified; time-activity curves showed that the tracer uptake in infected mice was significantly higher than mice without infections; (d) the average tracer uptake in the brain from 30 to 50 min of the PET scan showed a dose-dependent manner. Data represent the mean ± SEM, *n* = 3 for each group.

S1PR1-specific radiotracer [^18^F]TZ4877 was used in this study in order to detect changes of S1PR1 in response to *S. aureus* infection. The synthesis and quality control of [^18^F]TZ4877 were achieved as previously reported (Figure 1(a)) [33]. For each batch, the synthesis of [^18^F]TZ4877 was accomplished with a radiochemical purity of >99%, chemical purity of >95%, and molar activity of >48 GBq/*μ*mol (decay corrected to the end of synthesis, EOB).

### 2.3. Bacterial Cell Culture

*Staphylococcus aureus* (ATCC 29213) was purchased from the American Type Culture Collection. The stock *S. aureus* was plated on LB agar plates overnight; individual colony was picked from LB agar plate and cultured in Trypticase Soy Broth at 37°C and 210 rpm until approximately 10 × 10^8^/mL of bacterial concentration was reached. The bacterial concentration was measured using a DEN-1 densitometer (Grant Instruments, UK). *S. aureus* was then centrifuged and resuspended in 25 *μ*L of PBS at a colony-forming unit (CFU) of 1 × 10^8^ CFU of live bacteria for high titer and 1 × 10^6^ CFU of live bacteria for low titer and used immediately.

### 2.4. S. aureus Infection through Local Inoculation

All experimental procedures involving animals were performed according to guidelines established by the Animal Studies Committee at Washington University in St. Louis. Seven-week-old Balb/c mice (Charles River, Wilmington, MA) were used in all studies. All animals were randomly assigned to each study group. In order to evaluate the effect of *S. aureus* infection on S1PR1, a bacterial infection model was created following the previously described procedure [36–38]. Previous studies demonstrated that S1PR1 is significantly increased 24 hours after Newcastle disease virus infection; in order to detect the change of S1PR1 expression using PET imaging, a relatively high dose of *S. aureus* and 24-hour treatment were used for initial evaluation of the response of S1PR1 to the bacterial infection. In fact, it has been reported that 10 [7] to 10 [9] CFU *S. aureus* can cause bacteria to elicit inflammatory responses within 24 hours [39–41], and intravenous inoculation of 5 × 10^7^ to 5 × 10^8^ CFU *S. aureus* can cause the animal to develop septic shock within 48 hours [42]. In our case, 1 × 10^8^ CFU (high titer) and 1 × 10^6^ CFU (low titer) of live *S. aureus* were diluted in 25 *μ*L PBS and injected in the muscle of the lower hind limb 24 hours prior to the study; 25 *μ*L of sterilized PBS was also injected to the contralateral limb as an internal control; in the meantime, a group of sham animals received the same volume of sterile PBS. For blocking studies, either S1PR1-specific ligand NIBR-0213 (Sigma-Aldrich, St. Louis, MO) at a dose of 5 mg/kg or S1PR1-specific DsiRNA (mm.Ri.S1pr1.13, Integrated DNA Technologies, Coralville, IA) at a dose of 20 *μ*g/kg was administered 12 hours prior to injection of radiotracer, noticing that NIBR-0213 has a long elimination half-life and prolonged duration of action in rodent [43]. In addition, our initial pilot test demonstrated that NIBR-0213 can successfully block our radiotracer long time after treatment.

### 2.5. Ex Vivo Biodistribution Studies

To characterize the distribution and kinetics of [^18^F]TZ4877 in different tissues in normal mice, we performed an ex vivo biodistribution study in normal Balb/c mice as previously described with minor modifications [33]. In brief, approximately 2.2 MBq/100 *μ*L of the radiotracer was administrated to the mice intravenously via the tail vein. At 5, 30, or 60 min posttracer injection, animals were euthanized and tissues of interest including the blood, lung, liver, spleen, kidney, muscle, heart, brain, pancreas, thymus, and small intestine were collected, weighed, and counted in a Beckman 8000 automated gamma counter (Beckman, Brea, CA). The radiotracer uptake in each tissue was calculated as background and decay-corrected percent injected dose per gram (%ID/g).

To determine the radiotracer uptake in different tissues in response to *S. aureus* infection, we then performed an ex vivo biodistribution study in normal and *S. aureus*-infected Balb/c mice. After 24 hours of infection through local inoculation, biodistribution study was performed as described above, tissues of interest from sham and infected mice were collected 30 min posttracer injection, and the uptake in each tissue was calculated. To determine if the increased uptake of [^18^F]TZ4877 was caused by endogenous S1PR1 activation, the infected mice were pretreated with an S1PR1-specific antagonist, NIBR-0213 [43], at 5 mg/kg and 12 hours prior to tracer injection. In addition, we used S1PR1-specific DsiRNA at 20 *μ*g/kg pretreatment in mice 12 hours prior to tracer injection to further confirm that [^18^F]TZ4877 is specific to S1PR1 *in vivo*, rather than other S1P receptor subtypes. The infected mice received 1 × 10^6^ CFU of live *S. aureus* that was diluted in 25 *μ*L PBS and injected into the muscle of the lower hind limb 24 hours prior to the study.

### 2.6. Immunohistochemistry

To confirm the increased uptake of [^18^F]TZ4877 in *S. aureus*-infected mice is caused by the upregulation of S1PR1, immunohistochemistry staining was performed in hind limb muscle from sham and *S. aureus*-infected mice. 14-micron sections from fresh frozen tissue were used. Sections were prewarmed to room temperature and fixed with 4% paraformaldehyde in PBS for 15 minutes and then washed in PBS. Sections were then incubated in Antigen Retrieval Buffer (Abcam, Cambridge, MA) for 20 minutes in boiling water bath and then blocked with 5% horse serum for 2 hours at room temperature followed by blocking with ReadyProbes Endogenous HRP and AP Blocking Solution (ThermoFisher, Waltham, MA). After washing in PBS, all sections were then incubated with rabbit anti-S1PR1 (Alomone, Israel) antibody overnight at 4°C and then washed and incubated with ImmPRESS HRP Horse anti-rabbit polymer for 1 hour at room temperature and developed with ImmPACT DAB (Vector Laboratories, Burlingame, CA).

### 2.7. MicroPET/CT Studies

To confirm the changes of S1PR1 expression in response to *S. aureus* inoculation, as well as to evaluate the feasibility of our S1PR1-specific radiotracer in detecting the infection-induced S1PR1 activation, microPET/CT studies of [^18^F]TZ4877 were performed in normal controls and *S. aureus*-infected mice. For the microPET studies, an Inveon MM PET/CT scanner (Siemens, Germany) was used; mice were anesthetized with 2% isoflurane under gas anesthesia during the imaging data collection period. Mice were secured using a custom-designed acrylic restraining device. Following a transmission scan and a computed tomography for anatomical registration, 6.8-7.6 MBq/mouse of [^18^F]TZ4877 was administrated via the tail vein using a catheter for injection. A list-mod protocol was used with 60-minute dynamic data acquisition with a dynamic sequence of 1 × 3 s, 6 × 2 s, 9 × 5 s, 6 × 10 s, 4 × 30 s, 2 × 1 min, 2 × 2 min, and 10 × 5 min frames. PET image data was processed and analyzed using Inveon Research Workstation software IRW 4.2 (Siemens, Germany). The data was reconstructed per time frame using an interactive reconstruction algorithm and corrected for decay. The tissue uptake of the radioactivity was measured from elliptical ROIs, and percent injected dose per gram of tissue (%ID/g) was calculated. To determine if the uptake of [^18^F]TZ4877 was correlated with the severity of the infection, microPET studies were also performed in mice that were injected with a high dose of live *S. aureus* (1 × 10^8^ CFU of live *S. aureus*).

### 2.8. Statistical Analysis

All data were analyzed with Prism 7.0 (GraphPad Software, San Diego, CA). Two-way ANOVA and paired Student *t*-test were used for comparison of percent injected dose per gram of tissue from ROI for PET study; two-way ANOVA with a relatively powerful Fisher LSD multiple comparisons test was used for comparison of percent injected dose per gram of tissue from each organ among different sample groups for the tissue distribution analysis. A *P* value ≤ 0.05 was considered to be statistically significant.

## 3. Results

### 3.1. Biodistribution Studies in Normal and S. aureus-Infected Mice

We first evaluated the kinetics of [^18^F]TZ4877 in mice at 5, 30, and 60 min postinjection (Table 1). The initial tracer uptake was high in tissues at 5 min; a rapid clearance of radioactivity was observed in the majority of tested tissues including the blood, lung, liver, spleen, kidney, muscle, heart, brain, pancreas, and thymus from 5 min to 60 min. Tracer uptake gradually accumulated in the small intestine, suggesting it had hepatobiliary clearance. In general, at 30 min postinjection in normal Balb/c mice, the muscle had the lowest tracer uptake among the tissues evaluated with a %ID/g of 1.4 ± 0.18, whereas the mouse liver and small intestine had the highest tracer uptake level with %ID/g of 15.27 ± 0.63 and 11.73 ± 0.59. Notably, [^18^F]TZ4877 showed a relatively high mouse brain uptake with %ID/g of 4.15 ± 0.19 at 30 min postinjection, indicating that [^18^F]TZ4877 penetrates the blood-brain barrier well.

We next compared the tissue uptake of [^18^F]TZ4877 between normal and *S. aureus*-infected mice. Interestingly, in response to *S. aureus* infection, an increase uptake of [^18^F]TZ4877 in several tissues was observed. After 30 min postinjection, the uptake of [^18^F]TZ4877 in serval organs including the lung, liver, spleen, brain, heart, kidney, thyroid, pancreas, thymus, and small intestine of infected mice was higher than that of sham mice (Table 2; ANOVA: *F* (1, 60) = 54.6, *P* < 0.0001). Fisher's LSD test following ANOVA showed that the tracer uptake in several tissues was significantly higher in mice with infections including the liver (*P* = 0.002; 30.6% of increase), kidney (*P* = 0.0031; 54.3% of increase), pancreas (*P* = 0.0027; 74.3% of increase), thymus (*P* = 0.0006; 115.3% of increase), and small intestine (*P* < 0.0001; 40.0% of increase).

In addition, the *S. aureus*-induced increase uptake of [^18^F]TZ4877 can be blocked by NIBR-0213 pretreatment and S1PR1-specific DsiRNA pretreatment. In general, NIBR-0213 significantly reduced the uptake of [^18^F]TZ4877 in several organs of the infected mice (Table 2; ANOVA between infected vs. infected with NIBR-0213: *F* (1, 60) = 22.7, *P* < 0.0001). Fisher's LSD test following ANOVA showed that the uptake of [^18^F]TZ4877 was significantly decreased after NIBR-0213 pretreatment in the liver (*P* = 0.0002; 22.5% of decrease), kidney (*P* = 0.0225; 30.7% of decrease), and small intestine (*P* = 0.0011; 12.0% of decrease). Furthermore, similar to the treatment with S1PR1 antagonist, S1PR1-specific DsiRNA also significantly reduced the tracer uptake in the liver and small intestine of the infected mice to the uptake level of [^18^F]TZ4877 in sham mice (Table 2; ANOVA between infected vs. infected with DsiRNA: *F* (1, 60) = 15.24, *P* = 0.0002). Fisher's LSD test following ANOVA showed that the tracer uptake was significantly restored after DsiRNA treatment in the liver (*P* = 0.0035; 15.4% of decrease) and small intestine (*P* < 0.0001; 7.8% of decrease).

### 3.2. MicroPET Studies in Normal and S. aureus-Infected Mice

*In vivo* microPET studies were carried out in three groups of mice, a low-dose inoculation of bacteria (1 × 10^6^ CFU), a high-dose inoculation of bacteria (1 × 10^8^ CFU), and sterile PBS buffer. Similar to biodistribution results, a systemic increased uptake of [^18^F]TZ4877 was identified in microPET studies. The radiotracer uptake in the liver and brain, as well as several other tissues, was significantly increased in the infected mice. Interestingly, the uptake of [^18^F]TZ4877 in these organs showed a dose-dependent manner to the *S. aureus* infection (Figure 1(b), Supplemental Figures 1 and 3). For example, tissue time-activity curves showed that the brain uptake in the infected mice was significantly higher than the uptake in sham mice in a dose-dependent manner (Figure 1(c); ANOVA test: high vs. low: *F*(1,120) = 9.62, *P* = 0.0024; high vs. sham: *F*(1, 80) = 95.6, *P* < 0.001; low vs. sham: *F*(1,120) = 337.2, *P* < 0.001). Comparing with sham mice, though the difference between high and low dose of infection was not statistically different, the uptake of [^18^F]TZ4877 in infected mice from 30 to 50 min of the scan was ~49.4% higher in the high-dose group and ~34.8% higher in the low-dose group (Figure 1(d)).

**Figure 2:**
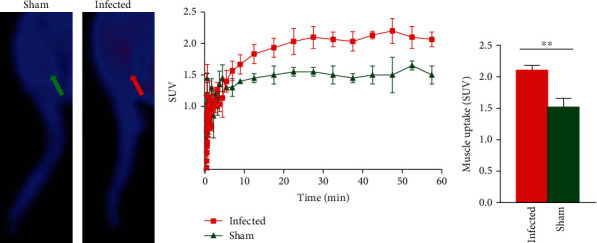
MicroPET imaging of S1PR1 activity in *S aureus*-infected mice. (a) Representative sagittal microPET images of [^18^F]TZ4877 in the hind limb of mice. The tracer uptake was relatively low in the hind limb muscle with a SUV of ~1.5 in sham mice. Comparing with sham mice, the tracer uptake was significantly higher in the hind limb of infected mice; (b) time-activity curves showed that the tracer uptake in infected mice was significantly higher than sham mice; (c) the average tracer uptake in the hind limb muscle from 30 to 50 min of the PET scan showed a ~39% increase of SUV in infected mice with a *P* value of 0.0082. Data represent the mean ± SEM, *n* = 3 for each group.

MicroPET study showed that the uptake of [^18^F]TZ4877 in the hind limb muscle was relatively low with an SUV of ~1.5 in sham mice. Interestingly, though the uptake of [^18^F]TZ4877 was low, compared with sham mice, the uptake of [^18^F]TZ4877 in the hind limb of *S. aureus*-infected mice was significantly higher than that of the sham mice. The average tracer uptake in the hind limb muscle from 30 to 50 min of the PET scan showed a ~39% increase uptake (SUV) of [^18^F]TZ4877 in infected mice compared to that in the normal control mice with a *P* value of 0.0083 (Figures 2(a)–2(c)). Notably, the increase of tracer uptake in the muscle was higher than the increase in the brain and liver of the infected mice (Table 3). Moreover, the increase of [^18^F]TZ4877 uptake was only identified in the ipsilateral side of the infection site but not in the contralateral site (Supplemental Figures 2 and 3). In contrast, after 24 hours of inoculation, the expression of S1PR1 measured by PET with [^18^F]TZ4877 showed almost no uptake in the hind limb muscle of the high-dose inoculation group (Supplemental Figure 1).

### 3.3. Immunohistochemistry Analysis in S. aureus-Infected Mice

**Figure 3:**
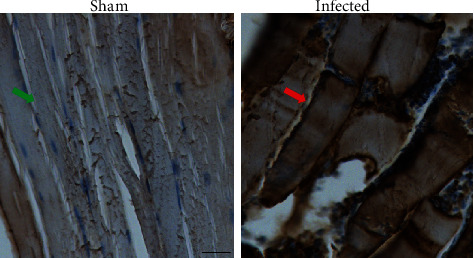
Immunohistochemistry analysis of S1PR1 in hind limb muscle of sham and *S aureus*-infected mice. S1PR1 was significantly upregulated in the muscle of infected mice (red arrow) comparing with sham mice (green arrow), scale bar = 100 *μ*m.

Immunohistochemistry analysis was carried out in the hind limb muscle of mice with and without infection. After 24 hours of *S. aureus* infection, the expression level of S1PR1 was significantly elevated in the muscle of infected mice in agreement with the increased uptake of [^18^F]TZ4877 (Figure 3), indicating that the upregulation of S1PR1 was tightly correlated with the pathogen-derived inflammation.

## 4. Discussion

Increasing evidence indicates that S1PR1 expression is tightly correlated with the inflammatory response to infectious diseases such as viral infections [44–49], bacterial infections [50–55], protozoan infection [56], and fungal infection [57, 58]. It is believed that S1PR1 plays an important role in maintaining endothelial barrier function and integrity in normal as well as inflammatory conditions and can modulate the endothelium to suppress inflammatory responses [59].

Mouse models for infectious diseases caused by *S. aureus* have been widely used for the evaluation of *S. aureus* skin and soft tissue infection, bacteremia, sepsis, peritonitis, and endocarditis [3]. Immune-competent mice, such as C57/BL6 and Balb/c mice, are considered a good candidate for the study of *S. aureus* infection in soft tissue [3]. The primary aim of this study was to evaluate if there is a change of S1PR1 expression in response to *S. aureus* infection and if such infection-induced changes of S1PR1 can be detected by our S1PR1-specific radiotracer [^18^F]TZ4877. Therefore, a relatively high dose of *S. aureus*, 1 × 10^6^ CFU, was used to induce the infection, and an even higher dose at 1 × 10^8^ CFU was used to compare if the changes of S1PR1 were *S. aureus* dose dependent. In the meantime, in order to detect the local and systemic changes of S1PR1, a relatively long inoculation period of 24 hours was chosen. In fact, it has been well known that soft tissue infection with 10 [7] to 10 [9] CFU *S. aureus* can cause bacteria to elicit inflammatory responses within 24 hours [39, 40]; the abscess lesions can increase over 5 to 7 days after infection [40] and can be gradually revolved over 7 to 9 days [39, 60].

In this study, we report a change of S1PR1 expression in response to the local *S. aureus* after 24 hours of infection. We observed a local change of [^18^F]TZ4877 uptake at the bacterial infection site as well as increased uptake of [^18^F]TZ4877 throughout the body. In general, both biodistribution and microPET studies showed that [^18^F]TZ4877 had high uptake in the majority of tested organs indicating that PET [^18^F]TZ4877could be a reliable tool for quantifying S1PR1 *in vivo*. For example, at 30 min postradiotracer injection, the biodistribution data showed that the liver uptake was ~15.27 (%ID/g); microPET studies showed that the liver uptake was ~11.40 (SUV). Notably, [^18^F]TZ4877 entered the brain well with an SUV value of ~3.57 at 30 min posttracer injection, indicating that the tracer can penetrate the blood-brain barrier well and has a great potential for imaging S1PR1 in the central nervous system.

One of the major findings of this study is the upregulation of S1PR1 measured by [^18^F]TZ4877 throughout the body in response to infection. Remarkably, this infection-induced upregulation of S1PR1 showed an *S. aureus* dose-dependent manner. S1PR1 is an immune modulator and plays a crucial role in the regulation of cytokine during inflammation [59]. It is reported that S1PR1 regulates cytokine production and host innate immune responses to pathogen infection [24]. In fact, several studies demonstrated that S1PR1-specific ligands can downregulate and control the massive innate inflammatory response, and endogenous S1P-S1PR1 axis could be a negative regulator of cytokine production [25, 59, 61]. The mechanisms of S1PR1 in the regulation of immune responses in response to pathogen infection remain not clear; our microPET study demonstrates a systemic activation of S1PR1 in response to local *S. aureus* infection and provides an evidence of the role of S1PR1 in the innate inflammatory response. In agreement with our microPET study, biodistribution data also showed a statistically increased uptake of [^18^F]TZ4877 in liver, kidney, and other tissues. In fact, accumulated evidence suggests that S1PR1 activation and signaling are impaired during infectious disease. For example, previous studies report that influenza infection can alter the expression level of S1PR1 in the liver, spleen, kidney, and even heart [26]. Also, suppression of early innate immune responses through S1PR1 signaling can significantly reduce mortality during infection with influenza virus by suppressing the pathogen-induced excessive host immune response, in other words, “cytokine storm” [59, 62, 63]. Innate immune response plays as the first line of defense against infections. It is well known that S1P/S1PR pathways have modulatory effects in cytokine secretion of innate immune response. In particular, S1PR1 regulates cytokine secretion in various types of cells including dendritic cells, macrophages, T lymphocytes, epithelial cells, and endothelial cells [61]. It is suggested that targeting on S1P/SPRR may be an effective therapy for such cases where the host inflammatory response is a major component in the disease process [61]. Particularly, numerous studies have shown that acute lung injury is a common consequence of cytokine storm in Middle East respiratory syndrome (MERS) and SARS, as well as COVID-19. S1PR1 inhibitor analogs, such as AAL-R and RP-002, which have already shown a protective effect from the pathophysiological response during influenza infection, have been suggested as potential immunomodulators to suppress the cytokine storm. Our study identified a systemic “storm” of S1PR1 activation in the presence of pathogens; this finding may provide a new insight into the relationship between pathogen-induced S1PR1 activation and cytokine storm. However, it remains unclear whether the systemic activation of S1PR1 is the direct result of pathogen infection; further characterization is required to understand more about this global protein activation.

On the other hand, accumulated evidence also suggests a potential role of S1P/S1PR pathways in regulating sepsis [9–11, 23, 64]. It is hypothesized that the S1P/S1PR pathway impairs antimicrobial defense in the pathogenesis of sepsis. The major processes of sepsis include immunological stimulation, systemic inflammation, and coagulopathy; recent studies suggest that the S1P/S1PR1 pathway is involved in the hyperinflammatory phase in sepsis and regulates the excessive release of cytokine; activation of S1PR1 reduces the severe complications in sepsis [64]. In fact, *S. aureus* is a common cause of sepsis normally caused by the bacterial replication in blood. Intravenous inoculation of 5 × 10^7^ to 5 × 10^8^ CFU *S. aureus* can cause the animal to develop septic shock within 48 hours [42]. Though a mouse model for *S. aureus*-induced sepsis is usually introduced intravenously with up to 5 × 10^8^ CFU *S. aureus* [3], in our case, a relatively long infection time was used; the infected animal is possibly septic 24-hour postinoculation particularly in mice with high-dose infection. Overall, our study provides a direct evidence of S1PR1 activation in response to pathogen infection; our microPET study demonstrates the feasibility of imaging the infection-induced activation of S1PR1 using the S1PR1 specific tracer [^18^F]TZ4877. Future studies with different doses of *S. aureus* and different infection times are needed to understand the precise mechanisms of the *S. aureus* infection-induced activation of S1PR1.

In addition to the systemic activation of S1PR1, using microPET study with [^18^F]TZ4877, we also observed a local upregulation of S1PR1 in the muscle of the infection site but not the contralateral side of the muscle indicating that such upregulation of S1PR1 is tightly correlated with pathogen-derived inflammation. In fact, recent data suggest that bacterial infections may affect inflammatory processes in the vascular wall and atherosclerotic plaque progression in peripheral arteries [65]. S1P-S1PR1 axis is involved in host protective effects during the inflammation by maintaining vascular integrity [66]. It is believed that activation of S1PR1 in endothelial cells can maintain vascular integrity and prevent vascular leak during inflammatory response. In our case, the local upregulation of S1PR1 in an infected region could be related to the infection-triggered vascular leak and is the part of physiological compensatory response in the endothelial cells to prevent vascular leak during the inflammation. Moreover, S1P-S1PR1 axis also plays an important role in the control of skeletal muscle metabolism, atrophy, and regeneration. Previous studies reported that S1P-S1PR1 axis is involved in the migration of satellite cell in the injured local to improve skeletal muscle tissue repair and regeneration [67]; intramuscular S1PR1 is elevated in regenerating muscle fibers and mediates skeletal muscle mass and function [68]. The detected upregulation of S1PR1 in the infected region could be related to the changes of muscle metabolism and regeneration induced by pathogen infection; further investigation is needed to understand the precise role of S1PR1 in the muscle in response to pathogen infection. Although the uptake of [^18^F]TZ4877 is relatively low in the muscle, the uptake of [^18^F]TZ4877 in the muscle of infected mice was significantly higher than that in the muscle of the control mice. Due to the relatively lower muscle uptake even in the infected mice, it will be a challenge for PET imaging with [^18^F]TZ4877 for the precise assessment of S1PR1 changes in muscle in response to pathogen infection. However, PET imaging with [^18^F]TZ4877 could provide a noninvasive tool for detecting S1PR1 in response to infection in other organs, such as the spinal cord, brain, lung, or liver [33, 34].

One limitation of the current study is the use of PBS instead of heat-killed *S. aureus* in the contralateral side of infection as an internal control. Heat-killed *S. aureus* has been widely used as an internal control in a murine myositis model. In this study, we attempt to investigate the S1PR1 expression in response to *S. aureus* infection using our S1PR1-specific radioligand [^18^F]TZ4877. Previous studies have demonstrated that heat-killed *S. aureus* can induce a strong anti-inflammatory response via Toll-Like Receptor 2 (TLR2) pathway; it can induce the production of TNF-*α*, IL-6, and IL-10, as well as other cytokines and chemokines [69–71]. On the other hand, S1P receptors including S1PR1 can attenuate the TLR2 pathway [72, 73]. To date, the effect of heat-killed *S. aureus* on S1PR1 remains unclear. To minimize the potential effect of heat-killed *S. aureus* on S1PR1 expression, we used PBS as an internal control in this study. Sepsis is a systemic inflammatory disease resulting from pathogen infection and is associated with acute and chronic changes in the central nervous system, particularly at the blood-brain barrier (BBB) [74]. To detect the changes of S1PR1 expression in response to *S. aureus* infection, an animal was injected with *S. aureus* at doses of 1 × 10^6^ CFU and 1 × 10^8^ CFU, and a microPET study was performed post-injection of *S. aureus*. Although the increased S1PR1 expression at the hind limb of the animal in response to *S. aureus* was confirmed by immunohistochemistry study, the reason that caused the increased brain uptake of [^18^F]TZ4877 in response to *S. aureus* is not clear. The increased brain uptake of [^18^F]TZ4877 could be resulted from increased brain S1PR1 expression or caused by sepsis-induced BBB breakdown. In addition, the increased brain uptake of [^18^F]TZ4877 in response to *S. aureus* was based on the assumption that the injection dose of *S. aureus* and incubation time (24 hrs) did not cause the sepsis. Further study using different doses of *S. aureus* and incubation times is necessary to understand the exact mechanisms of the elevation of S1PR1 in response to *S. aureus* infection.

## 5. Conclusions

In summary, we investigated the expression of S1PR1 in *S. aureus*-infected mice using both ex vivo biodistribution studies and microPET imaging; our data suggested that the infection-induced systemic S1PR1 activation is tightly correlated with the early immune response to infection. The uptake of the S1PR1 radiotracer [^18^F]TZ4877 can be blocked by S1PR1-specific antagonist and S1PR1-specific DsiRNA. Further characterization of the mechanisms of infection-induced systemic S1PR1 activation and translational investigation of S1PR1 functions in infectious diseases may lead to a new strategy for the diagnosis and the treatment of infectious diseases.

## Figures and Tables

**Table 1:** Biodistribution (%ID/g, mean ± SEM) of S1PR1-specific [^18^F]TZ4877 in Balb/c mice (*n* = 4).

	5 min	30 min	60 min
Blood	3.78 ± 0.18	2.53 ± 0.08	2.68 ± 0.03
Lung	8.81 ± 0.44	5.8 ± 0.23	5.18 ± 0.08
Liver	20.07 ± 0.17	15.27 ± 0.63	17.29 ± 0.19
Spleen	4.94 ± 0.24	3.28 ± 0.17	2.92 ± 0.01
Kidney	10.47 ± 0.35	7.34 ± 0.45	6.57 ± 0.09
Muscle	1.76 ± 0.37	1.4 ± 0.18	1.53 ± 0.08
Heart	7.26 ± 0.26	4.39 ± 0.24	4.03 ± 0.13
Brain	4.96 ± 0.36	4.15 ± 0.19	3.78 ± 0.09
Pancreas	8.27 ± 0.51	5.42 ± 0.3	4.75 ± 0.19
Thymus	7.48 ± 0.68	5.61 ± 0.39	6.24 ± 0.56
Small intestine	8.06 ± 0.27	11.73 ± 0.59	17.92 ± 1.06

**Table 2:** Biodistribution of S1PR1-specific [^18^F]TZ4877 in sham, infected, and infected with treatments mice (*n* = 4).

	Sham	Infected	NIBR0213	siRNA
Blood	2.33 ± 0.17	1.96 ± 0.81	2.60 ± 0.1	2.46 ± 0.12
Lung	5.56 ± 0.18	6.68 ± 0.47	5.67 ± 0.22	5.57 ± 0.22
Liver	13.52 ± 1.29	17.65 ± 0.16^∗∗^	14.42 ± 0.46^###^	13.6 ± 0.21^##^
Spleen	2.39 ± 0.17	3.48 ± 0.19	3.17 ± 0.12	3.03 ± 0.11
Brain	3.36 ± 0.27	5.24 ± 0.12	4.55 ± 0.17	4.28 ± 0.13
Heart	3.56 ± 0.37	4.84 ± 0.16	4.20 ± 0.14	4.09 ± 0.19
Kidney	5.83 ± 0.39	8.99 ± 0.32^∗^	7.31 ± 0.18^#^	6.61 ± 0.27
Thyroid	2.83 ± 0.15	4.00 ± 0.12	3.29 ± 0.09	3.20 ± 0.25
Pancreas	4.33 ± 0.34	7.55 ± 0.54^∗^	6.10 ± 0.5	5.83 ± 0.85
Thymus	3.23 ± 0.18	6.94 ± 1.02^∗^	5.79 ± 0.76	5.94 ± 0.64
Small intestine	14.96 ± 1.12	20.94 ± 1.60^∗∗∗∗^	16.31 ± 2.13^##^	17.48 ± 1.73^###^

Data represents %ID/g and mean ± SEM, samples with a statistical difference were in italic; ^∗^Fisher's LSD multiple comparisons between sham and infected mice: ^∗^*P* < 0.05, ^∗∗^*P* < 0.01, and ^∗∗∗∗^*P* < 0.0001; ^#^Fisher's LSD multiple comparisons between infected and infected with NIBR0213 or infected and infected with siRNA: ^#^*P* < 0.05, ^##^*P* < 0.01, and ^###^*P* < 0.001.

**Table 3:** PET measurements of S1PR1-specific [^18^F]TZ4877 in *S aureus*-infected and sham mice.

	Infected^∗^	Sham^∗^	*P* value^#^	Changes (%)
Hind limb muscle	2.11 ± 0.04	1.52 ± 0.07	=0.0001	38.8%
Brain	3.57 ± 0.12	2.65 ± 0.14	=0.0082	34.8%
Liver	11.40 ± 0.20	10.13 ± 0.12	<0.0001	12.5%

^∗^ *N* = 3 per group, mean ± SEM of average SUV from 30 to 50 min. ^#^Two-tailed paired *t*-test.

## Data Availability

The data that support the findings of this study are available from the corresponding author upon request.

## Supplementary Materials

Supplementary Materials MicroPET/CT images of S1PR1 activity in sham and *S. aureus*-infected mice are provided as supplementary files. Supplementary Fig 1: representative sagittal, coronal, and transverse PET/CT images of [^18^F]TZ4877 in sham, low-dose infected, and high-dose infected mice. Supplementary Fig 2: representative PET and PET/CT images of [^18^F]TZ4877 in hind limb muscle of sham and *S aureus*-infected mice.
