# Supplementary material for: PET Study of Sphingosine-1-phosphate Receptor 1 Expression in Response to S. aureus Infection
Source: Mol Imaging. 2021 Oct 4;2021:9982020. doi: 10.1155/2021/9982020 (PMC8654346; doi:10.1155/2021/9982020)
Supplement: Supplementary Materials — MicroPET/CT images of S1PR1 activity in sham and S. aureus-infected mice are provided as supplementary files. Supplementary Fig 1: representative sagittal, coronal, and transverse PET/CT images of [18F]TZ4877 in sham, low-dose infected, and high-dose infected mice. Supplementary Fig 2: representative PET and PET/CT images of [18F]TZ4877 in hind limb muscle of sham and S aureus-infected mice. [file 9982020.f1.docx]

**Supplementary Material**

**PET Study of Sphingosine-1-Phosphate Receptor 1 Expression in Response to *S. aureus* Infection**

Hao Jiang^1^, Jiwei Gu^1^, Haiyang Zhao^1^, Sumit Joshi^1^, Joel S. Perlmutter^1,4^, Robert J. Gropler^1^, Robyn S. Klein^2,3,4^, Tammie L.S. Benzinger^1,5^, Zhude Tu^1*^

Table of Contents

[**Supplementary Fig. 1.** The uptake of [^18^F]TZ4877 in whole body of mice 2](#_Toc78450452)

[**Supplementary Fig. 2.** The uptake of [^18^F]TZ4877 in hind limb muscle of the mice. 3](#_Toc78450453)

[**Supplementary Fig. 3.** Reigons of interests for the brain and the hind limb muscle. 4](#_Toc78450454)


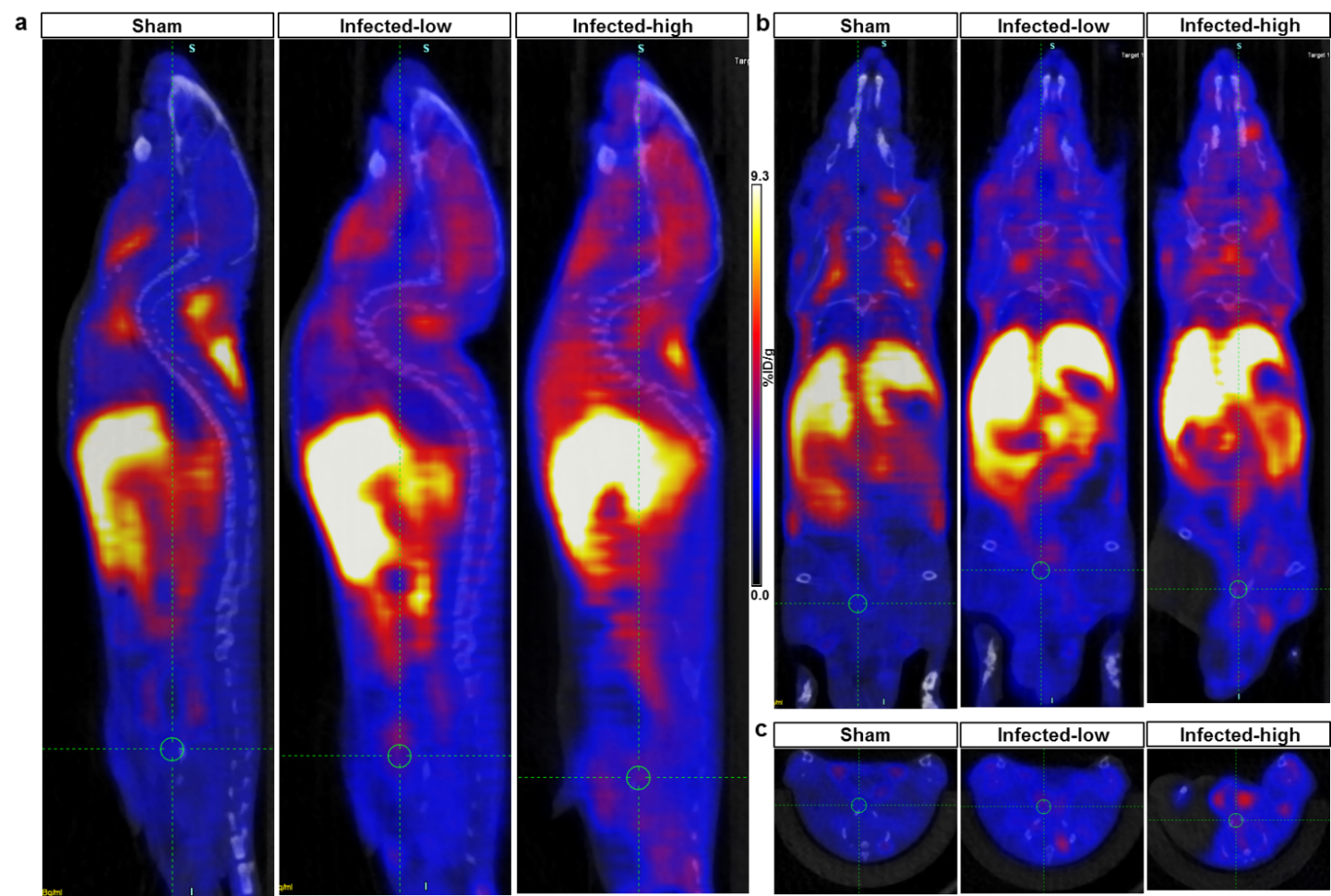


Supplementary Fig. 1. MicroPET/CT imaging of S1PR1 activity in sham and *S aureus* infected mice. Representative sagittal (a), coronal (b) and transverse (c) PET/CT images of S1PR1 specific radiotracer, [^18^F]TZ4877, in the same mice in Fig. 2a. Representative PET/CT images showed a systemic upregulation of S1PR1 in reponse to *S aureus* infection in a dose dependent manner.


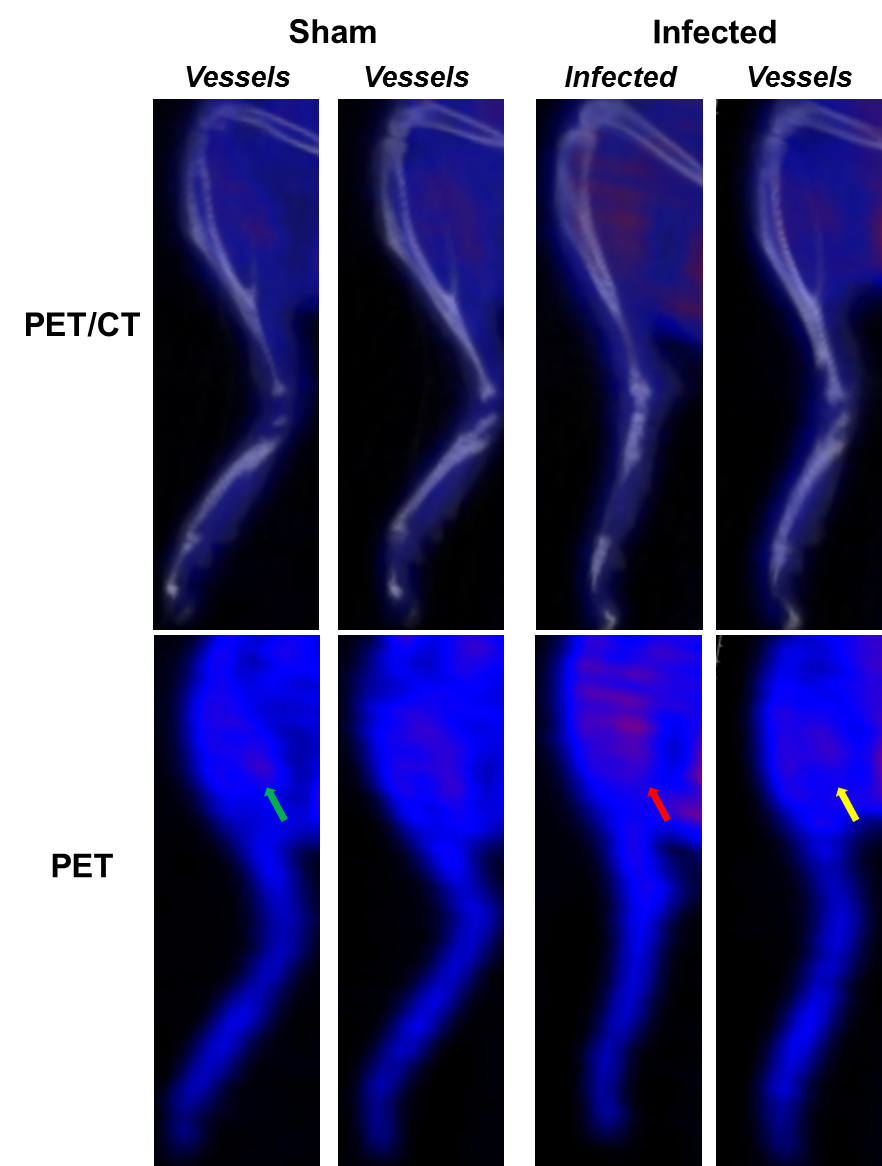


Supplementary Fig. 2. MicroPET/CT imaging of S1PR1 activity in hind limb muscle of *S aureus* sham and infected mice. Representative sagittal PET/CT images of S1PR1 specific radiotracer [^18^F]TZ4877 in the hind limb muscle of infected and sham mice. Comparing with sham mice (green arrow), a significant increase of tracer uptake was identified in the hind limb muscle of the infected mice (red arrow). In addition, the increased uptake of [^18^F]TZ4877 was only identified in the iplateral side of the infection but not in the muscle of contralateral side (yellow arrow), indicating the increased uptake of [^18^F]TZ4877 was a local effect in the infected site.


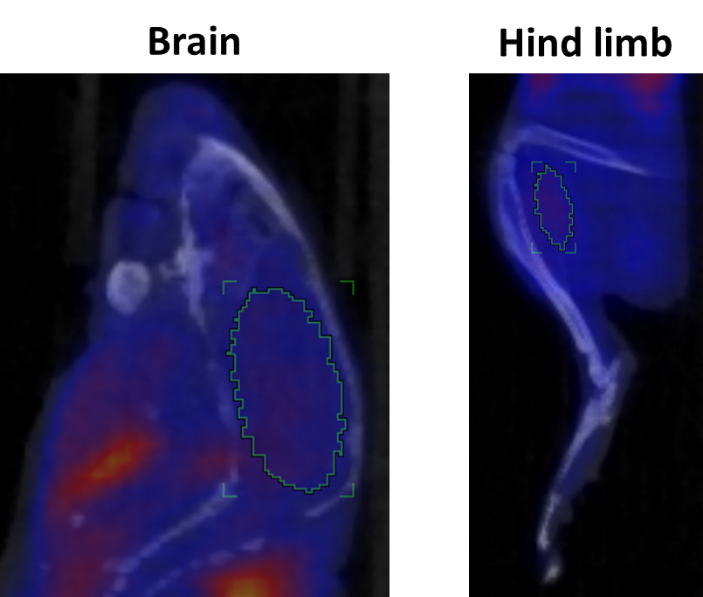


Supplementary Fig. 3. Representative ROI that used for quantification of [^18^F]TZ4877 in the brain and hind limb muscle.
